# Implementation of a national aging-in-place reform: a qualitative study exploring facilitators and barriers from the perspectives of employees in six Norwegian municipalities

**DOI:** 10.1186/s43058-025-00807-9

**Published:** 2025-11-13

**Authors:** Linda Aimée Hartford Kvæl

**Affiliations:** 1 Health Services Research Unit (HØKH), Akershus University Hospital, P.O. Box 1000, 1478 Lørenskog, Norway; 2 Research Centre for Habilitation and Rehabilitation Models and Services (CHARM), Institute of Health and Society, Faculty of Medicine, University of Oslo, Oslo, Norway

**Keywords:** Implementation, Aging in Place, Municipality Research, Older People, Quality Reform, Process Evaluation, Consolidated Framework for Implementation Research (CFIR), Norway

## Abstract

**Background:**

Despite many older individuals in Norway experiencing fulfilling lives, the effectiveness of local solutions for quality aging in place is inconsistent across different municipalities. To address this, White Paper No. 15, A Quality Reform for Older Adults, was introduced in Norway in 2019, targeting the challenges associated with aging in place and maintaining quality of life, irrespective of health status or location. The reform was based on recommendations from stakeholders: staff, older adults, relatives, volunteers, researchers, and leaders. This study explored how barriers, facilitators, and context interact in implementing a national aging-in-place reform and how these dynamics can inform actionable strategies for successful and context-sensitive implementation as experienced by municipal employees.

**Methods:**

This qualitative study, utilizing a process evaluation design and the Consolidated Framework of Implementation Research (CFIR), forms part of a larger reform evaluation. This study focuses on six Norwegian municipalities of varying sizes, from three counties in central, south, and north Norway, selected due to their focus on institutional or home care and their demographics. Data was collected through focus group discussions with municipal employees (*N* = 36), who represent a wide range of professional backgrounds and experiences.

**Results:**

The final analysis resulted in five main themes, structured in line with the CFIR framework: i) Policy Translation: Making Sense of the Reform in Local Context, ii) National Framing: Navigating Through Pandemic and Diversity, iii) Local Governance: The Need for a Common Implementation Platform, iv) Stakeholder Dynamics: The Importance of Interplay of Different Actors, and v) Sustainability Uncertainty: Lack of Clear Reform Responsibility. The themes provide an overview of facilitators and barriers during the reform implementation.

**Conclusions:**

The study found that despite municipalities’ diverse engagement with the reform’s focus areas, there is potential for more effective implementation. Municipal employees agreed with the reform’s ideas but struggled with its innovative aspects, indicating a need for clearer guidelines through top-down strategies. Facilitators were identified, but barriers such as the COVID-19 pandemic, municipal diversity, and funding issues created challenges. Insufficient leadership and inter-sector collaboration were primary obstacles. These findings are crucial for future reform implementation and service quality improvement.

Contribution to the literature

- Provides firsthand insights from municipal employees on the challenges of implementing an aging policy, framed within the CFIR framework to identify key barriers and facilitators
- Highlights the critical role of leadership responsibility and cross-sector collaboration in overcoming barriers and driving reform adoption.
- Stresses the importance of distinguishing a reform’s innovative aspects from existing practices to enhance implementation success.
- Underscores the role of dedicated champions in facilitating collaboration and ensuring reform integration in daily clinical practice.
- Emphasizes the need for tailored top-down strategies, such as leadership support, funding, and capacity-building, to complement local adaptations for sustainable implementation across contexts.

## Background

In Norway, like other Western countries, the ‘aging in place’ policy, which supports older individuals to age in their chosen homes and communities, has significantly impacted in recent decades due to an aging population [1]. From being a concept primarily defined as a contrast to aging in institutionalized settings [2], the concept has evolved and expanded to encompass both place and environment, support, technology, social networks, and personal characteristics [3]. Grasping the complexity of aging in place can explain why some older adults stay in their homes or communities or choose to move for aging-related preparation [4]. Indeed, the discussion today also implies aging in the ‘right’ place, emphasizing the importance of appropriate health and social care services, community support enabling full participation in later life, and thereby living one’s life with quality. Hence, Roger et al. (2020) describe aging in place as a process: «one’s journey to maintain independence in one’s place of residence as well as to participate in one’s community» ([5], p. 754).

A review of aging-in-place policy reforms in Europe identifies four key goals: expanding community-based care, increasing reliance on informal caregivers, implementing cost-saving measures, and improving access to quality care [6]. However, implementation varies due to differences in governance, resources, and cultural contexts [7]. In Norway, these policies align with international goals but face challenges from decentralized governance and geographic diversity, illustrating the concept's vagueness in addressing inequality dimensions [8, 9]. Consequently, social exclusion among older adults is often overlooked, and viewing them as a single category fails to address their diverse needs [10]. Research further highlights that age-friendly housing often benefits more privileged older adults [11], as those equipped with resources such as education, strong family ties, and high health literacy are better positioned to navigate healthcare complexities [12].

### The quality reform for older adults

To address these aging in place challenges, Norway introduced the ‘White Paper No. 15 (2019–2023) A Full Life – All Your Life: A Quality Reform for Older Adults’ in 2019. The reform promotes quality in later years by emphasizing autonomy, functionality, social participation, and access to healthcare. Moreover, the reform emphasizes a fulfilling life and aging in place, even as health deteriorates. The reform reflects core principles such as equity, dignity, independence, and user involvement, emphasizing aging in place for all, regardless of geography or socioeconomic status. These values guide the prioritization of barriers and strategies, ensuring tailored local plans and collaborative approaches.

The Quality Reform, based on input from employees, older adults, relatives, volunteers, researchers, and leaders, addresses five key areas where services for older adults often fall short: i) An age-friendly Norway: Encouraging older adults to plan their aging while municipalities create age-friendly communities and services in collaboration with public and private stakeholders, ii) Activity and community: Enhancing well-being and preventing health issues through tailored physical, social, and cultural activities that foster relationships and intergenerational connections, iii) Food and meals: Reducing malnutrition and promoting enjoyable mealtime experiences with nutritious, flexible, and welcoming meal options, iv) Healthcare: Providing tailored healthcare, involving older adults in decisions, and enabling them to live at home as long as possible with access to necessary care, and v) Continuity in services: Ensuring cohesive services for smoother transitions between home, hospitals, and care homes, while supporting caregivers and preventing burnout [13].

Implementation entail the systematic uptake of evidence-based knowledge into practice [14], improving the acceptance and long-term viability of a clinical program or new practice [15]. Implementation strategies should address barriers and facilitators [16, 17] and be tailored to local contexts [18]. The Quality Reform for older people is a complex intervention involving multiple components and personnel across organizations [19], relying primarily on bottom-up strategies led by municipalities. These include evaluating progress, identifying focus areas, and adopting locally tailored plans [13], aligned with ERIC (Expert Recommendations for Implementation Change) strategies like promoting adaptability, engaging stakeholders, and conducting needs assessments [20]. Top-down strategies, though fewer, involve national and regional support and mandating changes in the five key areas [13], aligning with ERIC strategies such as monitoring implementation and using advisory boards [20]. Despite lacking direct funding [13], the reform depends on local resourcefulness and collaboration, with financial being crucial for success [20]. Successful implementation is key to the reform`s effectiveness [15], requiring analysis of interventions like White Paper No. 15 [19, 21].

### Aim of the study

This study explored how barriers, facilitators, and context interact in implementing a national aging-in-place reform and how these dynamics can inform actionable strategies for successful and context-sensitive implementation as experienced by municipal employees.

## Methods

### Design

From a pragmatist standpoint [22], this qualitative study used a process evaluation design [23], as part of a larger reform evaluation [24]. The Quality Reform for Older Adults was assessed through registry data, surveys, document analyses, and interviews with regional staff, municipal leaders, administrators, and end-users in six municipalities. This study specifically analyzes focus group interviews with municipal employees.

### The consolidated framework for implementation research

In this study, the Consolidated Framework for Implementation Research (CFIR) was used as a theoretical guide. CFIR, widely referenced in implementation science, systematically examines implementation contexts through five domains: i) Intervention Characteristics, ii) Outer Setting, iii) Inner Setting, iv) Characteristics of Individuals, and v) Implementation Process. Drawing on 19 theories, CFIR identifies barriers and facilitators to intervention effectiveness and was updated in 2022 to reflect recent literature and user feedback [17, 25]. Its flexibility allows adaptation to various research designs and stages of implementation [26, 27]. CFIR was selected for its structured, context-sensitive approach, enabling a systematic evaluation of barriers and facilitators in complex interventions like the Quality Reform.

### Study setting

In Norway, health and social care services are mainly publicly funded and managed by 357 municipalities, leading to variations based on local resources, demographics, and geography. This study focuses on six municipalities of varying sizes, from three counties in central, south, and north Norway, selected due to their focus on institutional or home care and their demographics. Each county includes a municipality with an above-average proportion of residents aged 80 + receiving home care or living in nursing homes (Table 1).

**Table 1:** Overview of the included municipalities and counties

Number of Municipality	Context and Service Orientation	Land area and Density	Characteristics of the Population	Key Features
Municipality 1A	County A, Mid-Norway, Homecare-oriented	500–1.000 km^2^, < 10 per km^2^	5.000–10.000 inhabitants, 50–65% urban, Growth	Deficits in services
Municipality 1B	County B, North-Norway, Homecare-oriented	500–1.000 km^2^, < 10 per km^2^	Fewer than 3.000 inhabitants, 0–25% urban, Decline	High expenses
Municipality 1C	County C, South-Norway, Homecare-oriented	> 1.000 km^2^, < 10 per km^2^	Fewer than 3.000 inhabitants, 25–50% urban, Growth	High income
Municipality 2A	County A, Mid-Norway, Institutional-oriented	500–1.000 km^2^, 50–100 per km^2^	10.000–50.000 inhabitants, 65–100% urban, Growth	Economic strain
Municipality 2B	County B, North-Norway, Institutional-oriented	< 500 km^2^, < 10 per km^2^	Fewer than 3.000 inhabitants, 0–25% urban, Decline	High expenses
Municipality 2C	County C, South-Norway, Institutional-oriented	< 500 km^2^, 50–100 per km^2^	10.000–50.000 inhabitants, 65–100% urban, Growth	Increased debt

Table Explanation: Context and Service Orientation: Indicates the county, geographical region, and whether the municipality focuses on homecare or institutional services. Land area and Density: Refers to the total land area in square kilometers (km^2^) and the number of inhabitants per square kilometer (km^2^). Characteristics of the Population: Includes the total number of inhabitants, percentage living in urban areas, and whether the population is increasing, stable, or declining. Key Features: Highlights unique challenges or advantages, such as health service deficits, high expenses, economic strain, or increased debt

The municipalities differ in population trends, economy, and urbanization. While 2 A and 2 C are more populous cities with high densities and high urban populations, rural municipalities like 1B and 2B face population decline, lower densities, and low urban populations. Notably, 1 C, with the smallest population, has the largest land area and is the only municipality with high income. Other municipalities struggle with financial strain, high expenses, or debt. All municipalities require restructuring to address demographic and reform challenges.

### Participants

The participants comprised 36 municipal health and social care employees with diverse professional backgrounds and experiences, who are publicly employed and represent frontline providers implementing policies at the point of care. The average age of the participants was 47 years, with ages spanning from 24 to 67 years. Close to 75% of the participants had higher education degrees, and on average, the participants had been in their current role for 11 years, with a range of 1 to 29 years. The participants’ characteristics are displayed in Table 2.

**Table 2:** Characteristics of the municipal health and social care employees (*N* = 36)

**Age in years**	***n*** = 36
< 30	2
30–39	9
40–49	7
50–59	9
60–69	8
**Sex**	***n* = 36**
Male	4
Female	32
**Profession**	***n* = 36**
Registered nurse	12
Nursing assistant	7
Therapists	5
Social workers	6
Case managers	3
Physicians	3
**Education**	***n* = 36**
High School	8
Higher education (1–4 years)	16
Higher education (4 years +)	12
**Municipality**	***n* = 36**
1A	8
2A	6
1B	5
2B	5
1C	7
2C	5
**Experience (in years)**	*n* = 32* (*4 did not respond)
< 5	11
6–15	13
16–25	7
26-30	1

Among the nurses (*N* = 12), several held specialised roles in dementia and psychiatry, and six had leadership responsibilities at the team or department level. The physicians (*N* = 3) occupied senior positions, with two working in the municipality and one in a nursing home. Case managers (*N* = 3) allocated services based on the principle of the lowest possible level of care. The strategic sample also included nursing assistants, therapists, and social workers, representing a diverse range of frontline providers.

### Data collection

One focus group interview was conducted in each of the six municipalities (*N* = 6). These interviews included a mix of professions, sectors, and services, and the number of participants in each group ranged from 5 to 8 participants. The recruitment of employees for focus group discussions was facilitated by a key contact person in the six municipalities. The focus group discussions were conducted in person between February and June 2022.

Three researchers, each with extensive experience in clinical and/or research fields related to municipal healthcare, were responsible for data collection. In addition to leading the focus interviews, the team engaged in ongoing discussions to reflect on emerging themes, challenge assumptions, and ensure consistency throughout the data collection process and in writing the Norwegian evaluation report [24]. To ensure consistency, a semi-structured interview guide was used, covering themes such as i) understanding and knowledge of the reform, ii) the five focus areas (age-friendly environments, activity and community, food and meals, healthcare, continuity in services), iii) the integration and implementation of the reform, iv) tailoring to local context, and v) organizational structures and support. Focus group discussions lasted between 60 and 75 min. All discussions were audio-recorded and securely stored on the Services for Sensitive Data platform. Verbatim transcriptions of the recordings were done by an external party, in accordance with privacy guidelines.

### Data analysis

This publication is based on the author’s independent reanalysis using the CFIR framework, building on data initially collected and analyzed by the research team. To analyze the data, the author utilized reflexive thematic analysis grounded in Braun and Clarke’s experiential orientation [28]. The author thoroughly went through all transcripts, taking notes along the way, and then applied HyperResearch software for coding. The codes were grouped under the updated CFIR constructs, offering a structured framework to identify and interpret barriers and facilitators within different implementation contexts (Table 3).

**Table 3:** Example of the coding process

Quote	Code	Group	CFIR construct
«*A reform of this nature could push us to think outside the box—beyond health and care. If we can restructure the services creatively, we might garner support from sectors different from those we have been involved with before*» (2A)	- Beyond care collective - Creative restructures	Collaborative sector practice	Inner setting

Through comparison and development using the quotes and the entire dataset, the groups were reassessed, refined, and combined into five main themes. Participant selection followed Malterud's information power principle, considering study objectives, sample specificity, proven theory, dialogue quality, and analytical approach [29]. The principle of reflexivity played a crucial role [28] with the author presuming that implementing new reforms is a complex process often underestimated in required support and financing. The coding for this publication was conducted by a single author following Braun and Clarke’s methodology, with reflexive memos documented throughout. Findings were critically discussed and refined with research colleagues to ensure validity. To enhance credibility, the manuscript adheres to the Standards for Reporting Qualitative Research guidelines [30].

## Results

**Fig. 1:**
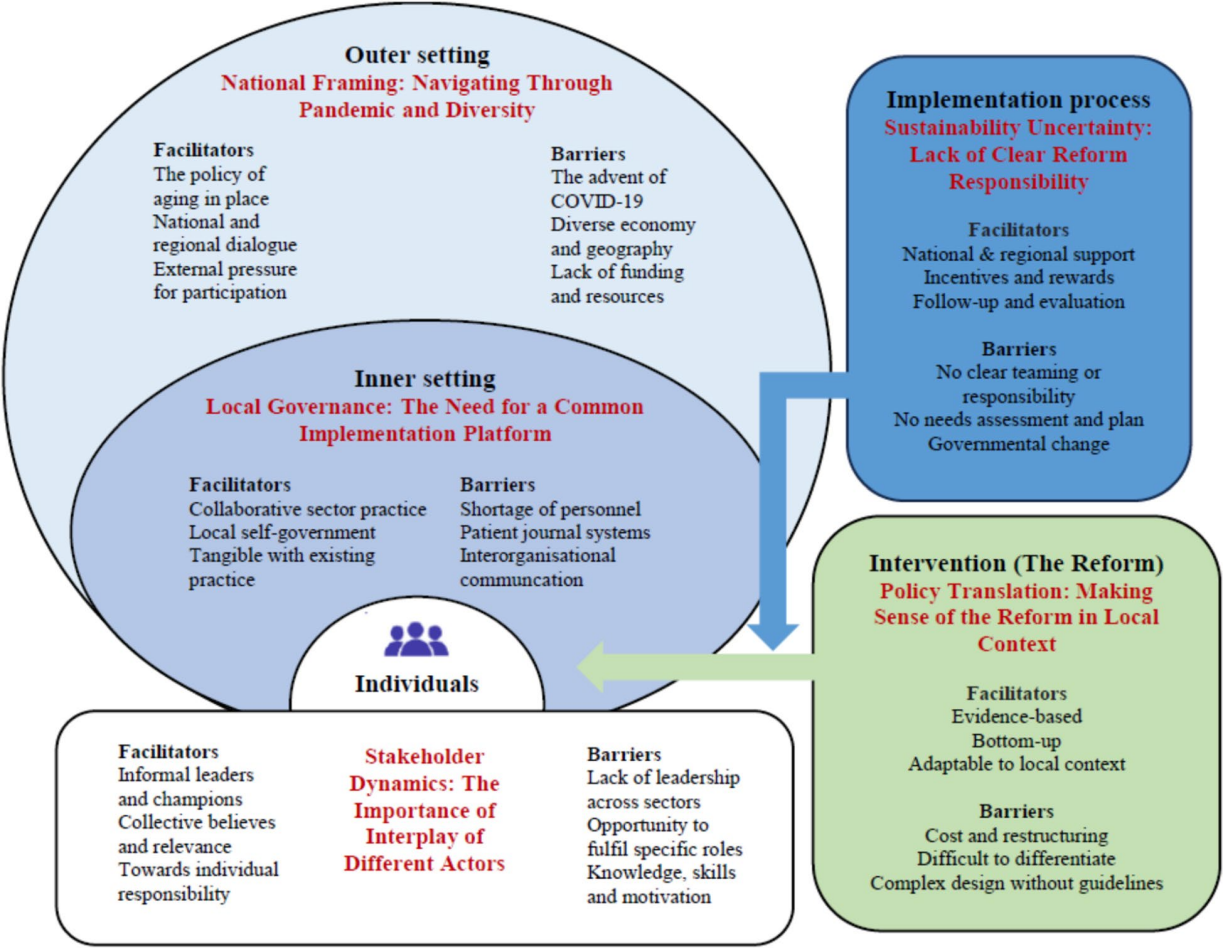
The interplay of themes with identified facilitators and barriers

The final analysis resulted in five themes structured in accordance with CFIR (see Fig. 1): i) Policy Translation: Making Sense of the Reform in Local Context, ii) National Framing: Navigating Through Pandemic and Diversity, iii) Local Governance: The Need for a Common Implementation Platform, iv) Stakeholder Dynamics: The Importance of Interplay of Different Actors, and v) Sustainability Uncertainty: Lack of Clear Reform Responsibility.

### Policy Translation: making sense of the reform in local context

The first theme, falling under the 'intervention characteristics' within the CFIR framework, focuses on how the reform is interpreted and applied within specific local contexts.

The municipal employees perceived the reform’s five focus areas positively. They regarded the Ministry of Health and Care Services as a credible source of innovation. All the proposed measures in the reform were perceived as robust proposals, substantiated by evidence supporting their effectiveness. However, a common barrier across all six municipalities was difficulty identifying what was truly new or innovative, as the focus areas had been longstanding priorities. Many questioned whether this should be labeled as 'reform work.'

«*I feel it's a bit like.... a lot of what we already do aligns with the reform’s focus areas, but that doesn't mean... Well, we might not have labelled it as reform work. Because these are things that have been happening and developing over years, and long before I came into the picture as well*» (1A)

Employees emphasized that the reform, developed through dialogue with older adults, staff, researchers, and other stakeholders, was relevant and adaptable to their municipalities. They agreed this flexibility was crucial, as municipalities had different priorities and had focused on various aspects of the reform. However, despite its adaptability, the need for restructuring to meet the focus areas was seen as a barrier due to increased costs.

«*Our municipality comprises numerous districts without a central hub for services. Transport poses significant challenges. For instance, we're unable to offer daily home nursing on isolated islands due to lack of services and staff. Home nursing six times a day isn’t common here, making a 5–10 year stay in an institution typical*» (1B)

While the focus areas were agreed upon, many employees, especially frontline staff like nursing assistants and social workers, lacked a clear understanding of the reform’s core message. Participants highlighted the need for a structured preparation phase with clear leadership roles, information meetings, and workshops to align communication across staff levels. The information deficit many experienced underscores the importance of effectively presenting complex innovations to ensure successful implementation.

«*We've heard about the reform for years. But as a clinician, I've not read it fully. I guess, those in planning likely used it more. We were actually scheduled for an information session that got repeatedly cancelled. Thus, we probably missed crucial joint information on the reform*» (2A)

In summary, while the reform was broadly agreed upon, challenges emerged in distinguishing its innovations and restructuring needs. Larger municipalities adapted more easily, while smaller ones faced resource and geographical constraints, such as remote service delivery. This highlights the need for clear guidelines and communication in the preparation phase.

### National framing: navigating through pandemic and diversity

The second theme corresponds with the CFIR construct 'outer setting' and highlights the broader, external factors that influenced the process and outcomes of the reform.

Municipal employees felt external pressure to join the national reform, as it was expected by other municipalities. Many noted that the reform’s focus areas aligned with the aging-in-place policy, supporting efforts to address future demographic trends, and highlighting its relevance. However, the reform was often described as a 'do-it-yourself' initiative due to the lack of funding and incentives, which posed a barrier in an already hectic work environment.

«*We aim to create a good society and care for all residents, a task becoming more challenging without sufficient labor or funds. Even if we had money, we lack people to hire. This ongoing struggle influenced my flippant nickname for the reform, which offers no funds or clear guidelines: the "do it yourself" reform*» (2C).

Participants identified COVID-19 in 2020 as a critical incident that delayed the reform’s rollout in municipalities. They explained that priorities shifted to vaccination and emergency preparedness, putting reform work on hold. Despite service disruptions and periodic lockdowns, employees felt the municipalities managed the situation effectively.

«*The COVID-19 pandemic has severely affected the provision of physical and social activities for older residents in the municipality. It's only now that we're starting to resume much of this work. Even though the municipalities did their best, it's only this week that we've stopped wearing masks at work*» (2B).

Municipal employees emphasized challenges in implementing the reform due to geographic and economic diversity across municipalities. While a national and regional support system was established to help, many staff were unaware of it. Key issues included unsustainable home services in remote island districts, limited age-friendly housing, and difficulties with transport and recruiting healthcare personnel.

«*We really need some sort of transportation to bring the older population into the town center. But so far, it hasn't been possible, and there are no activities in the outskirts. This situation leads to loneliness and sedentary lifestyles, and older individuals who are dependent on sedatives. I believe it's a significant problem*» (1C).

To summarize, while policy alignment and external pressures acted as enablers, the implications of the COVID-19 pandemic, diverse municipal challenges, and lack of funding incentives posed barriers in the reform's implementation journey. Smaller municipalities reported greater difficulties in maintaining home services and addressing transport needs.

### Local governance: the need for a common implementation platform

The third theme, which aligns with the CFIR 'inner setting', encompasses the environment in which the reform is being implemented, thereby affecting the implementation process.

All municipalities except two had handed the responsibility of implementing the reform solely to the health and care sector. This single-sector responsibility was described as vulnerable. However, two of the larger municipalities, benefiting from larger administrative structures and more resources, and a clear vision for broader collaboration, managed to demonstrate greater collaboration between sectors. In this way, the reform was more broadly grounded across sectors and established as a collaborative practice. Among all the municipalities, this was considered a strength, enhancing the integration of the reform work into the system.

«*I believe there's scope for improvement in cross-sector collaboration. We're operating in silos with health and care on one side, and community development, volunteering, and culture on another. We might be competent at an individual level, but I feel there's a lack in overarching coordination. We could gain more with better planning and more targeted efforts*» (2A).

Even though it could be challenging to differentiate the innovation aspect of the reform, it was still considered compatible with existing practice by the municipal employees interviewed, as the key areas were mostly already part of the practice. Apart from one financially stable municipality which experienced good access to health sector personnel, the lack of adequate personnel was a significant structural barrier to the implementation of the reform. This was a major concern particularly for smaller municipalities experiencing depopulation.

«*Sometimes, when resources are limited and staffing is weak, we actually don't have the capacity to utilize aids we could have used or initiate measures that could save resources in the long run. This leads to frustration. There's no scope to take the time needed to properly initiate and get things off the ground*» (2B).

The absence of a uniform electronic patient record system disrupted the continuity of care between hospitals, homes, and GPs. Additionally, employees spotlighted the multitude of social and physical activities provided by the municipality and volunteer organizations. However, difficulties emerge as volunteer initiatives often function independently, without a system to consolidate services for older adults within the municipalities. This lack of inter-organizational communication was seen as a hindrance to reform efforts.

«*Regarding activities, there's no place to find out what's happening and when. You know, where I can take my mother-in-law for knitting, exercise, etc. There's a shortfall there, as many believe nothing is happening. But a lot is going on, so we need to improve at showcasing the collective services in the municipality. Moreover, there's a delay in communication and knowledge about available services*» (1A).

In summary, while local governance, practice compatibility, and sector collaboration were strengths, reform efforts faced challenges from staff shortages, inconsistent patient records, and weak inter-organizational communication. Larger municipalities benefited from stronger administrative capacity, enabling collaboration, while smaller ones struggled with staffing and infrastructure. One high-income power municipality, however, avoided these issues.

### Stakeholder dynamics: the importance of interplay of different actors

The fourth theme explores stakeholder dynamics in the reform, revealing how different actors affect its execution and results, fitting into CFIR’s ‘individual characteristics’.

Employees highlighted a notable lack of consistent cross-sector and cross-level municipal leadership across all municipalities. Responsibility was often assigned to one person, typically in the health sector, creating continuity and coordination issues if they left unexpectedly. Despite this, work aligned with the reform's five key areas was carried out, though employees credited dedicated champions rather than strategic leadership.

«*In terms of dementia, we operate a school for relatives, and we've established our own dementia-friendly rambling route and host a dementia café monthly. However, it's the dementia coordinator who deserves the recognition for all the work done in this field, it's not an outcome of the reform leadership*» (2C).

Municipal employees observed a shift, especially in smaller rural municipalities, where older individuals were expected to take more responsibility for their aging. Services shifted from care to proactive support, positioning the reform as an attitudinal campaign to increase older individuals' accountability.

«*I see the reform mainly as an attitude shift. Instead of overprotecting the older population, we should highlight their independence and ability to make decisions, regardless of age. Being old is not a pitiful state, and we should assist where possible. I've personally noticed a change, and perhaps in the society as well*» (1B).

With frail older individuals increasingly expected to live at home or being discharged from hospitals in poorer health, employees noted limited time and knowledge to fulfill their roles under the reform. Many staff lacked an activity- and rehabilitation-focused mindset, and insufficient competence enhancement in the reform’s key areas was seen as a barrier to implementation.

«*We strive to incorporate exercise into the daily care routines. However, initiating this can be difficult. This likely arises from the attitudes and knowledge of the staff. Even though variations in patient occupancy affect available opportunities, confronting attitudes among the staff is a challenge we need to address*» (1C).

In summary, dedicated champions and the shift to proactive services facilitated the reform, while barriers included insufficient leadership, employee time constraints, and limited competence enhancement. This shift toward independence was more pronounced in smaller rural municipalities, where older residents had traditionally relied more on municipal care.

### Sustainability uncertainty: lack of clear reform responsibility

The final theme encompasses the activities and strategies used to incorporate the reform into clinical practice, corresponding to the ‘implementation process’ within the CFIR framework.

While municipal employees were aware of regional seminars for knowledge sharing and annual awards for top performers in reform work, the responsibility for reform was typically held by higher-ups. As a result, those working directly with older individuals, responsible for grassroots-level reform implementation, had minimal connection to these activities and limited information was communicated to them.

«*Originally, the responsibility rested solely with our manager. When she left, her replacement took over. In retrospect, we, as clinicians, should have been more involved. It seemed she steered the ship, while we were busy with testing and vaccinating. We were updated during a staff meeting two years ago*» (2B).

Each municipality was expected to tailor the reform based on a needs and context assessment, but no one described this process. Some saw the reform as reinforcing existing practices, while others viewed it as a shift toward personal responsibility and sustainable services. These differing interpretations were attributed to a lack of local grounding, such as the absence of an implementation team with dedicated cross-sector funding.

«*I believe the reform needed better preparation, top-down and in teams. More knowledgeable individuals are needed for effective collaboration across all services and sectors. Currently, everyone seems to be doing their own thing, fighting to manage their daily tasks*» (1C).

The follow-up and outcome evaluation encouraged municipal employees to reflect on their progress, potentially serving as a facilitator. However, enthusiasm for the reform waned over time due to factors like COVID-19 and a government change already planning a new reform.

«*This reform indeed has many good ideas, but I've seen little of the accompanying measures. There was supposed to be regional support and national gatherings, but I've not seen these in practice. Then March 2020 halted most non-urgent plans. Now, we have a new government...»* (2C)

In summary, despite national and regional support, the main barrier to the reform implementation appears to be the lack of municipal collaboration across levels and sectors, compounded by the absence of leadership responsibility to lead the implementation process through all phases. The need for municipalities to interpret and translate the reform on their own has resulted in varying understandings and inconsistent implementation approaches.

## Discussion

Municipal employees agreed on the adaptable reform but met challenges distinguishing its innovations and implications, underlining the need for clear and actionable guidelines. These guidelines should clarify the reform's program theory, define roles and responsibilities, and support collaboration and leadership development while being adaptable to local contexts. Policy alignment and external pressures facilitated the process, yet the COVID-19 pandemic, diverse municipal challenges, and funding issues posed barriers. The reform acknowledged local governance, practice compatibility, and sector collaboration, but faced hurdles such as limited healthcare personnel, inconsistent patient records, and poor communication. While dedicated champions and proactive services assisted, competence enhancement and time constraints posed difficulties. Despite national and regional support, the main barrier was lack of leadership and insufficient teaming across municipal sectors, as highlighted by participants' emphasis on unclear roles, poor collaboration, and fragmented efforts.

### The reform

The participants in our study regarded the Ministry of Health and Care Services as a credible source of innovation. As noted in the CFIR, the credibility of the source can be a key determinant in implementation processes, which potentially increases local investment [25]. The participants valued the bottom-up examples the reform provided, framed by evidence. Academic literature highlights research, clinical experience, and patient input as key to uptake [31]. Since the focus areas were longstanding priorities, participants struggled to see what was truly new, posing a barrier to implementation. This reflects an innovation’s relative advantage [17], which must be clearly demonstrated to convey its benefits and ease adoption [32].

Participants appreciated the reform's bottom-up approach but noted a lack of information and guidelines to support implementation. The complexity of an innovation increases when it targets multiple groups [33], making it essential to clearly define all components and provide tailored training materials and information across all levels of the care system, as highlighted in the CFIR [17]. Municipal differences in demography, geography, and resources influenced how the reform was interpreted, leadership structures, and cross-sector collaboration. These factors also impacted resource availability, including personnel, transport, housing, and activities, highlighting the need for local tailoring. Adaptable innovations correlate with successful implementation [34], but despite the reform's flexibility, restructuring and costs remained barriers, as noted in the literature [35].

### The outer setting

Participants highlighted the reform’s nationwide scope and focus on aging in place for sustainable health services as key motivators. These factors may serve as external pressures driving innovation delivery [36], linked to the CFIR outer setting [37]. Despite a national and regional support system, participants saw insufficient community-level funding as a major barrier to implementation. Research shows that reform implementation imposes significant financial burdens on municipalities due to activities beyond standard services, leading to both direct and indirect costs. Additionally, sustained support, particularly to address issues like clinician turnover, requires substantial ongoing investment [38].

All participants underscored the impact of COVID-19's emergence in 2020, which coincided with the scheduled rollout of the reform. They elaborated on how the pandemic required prioritizing vaccination and emergency preparedness over reform initiatives. As identified in CFIR-based studies, unexpected events, such as pandemics, weather-related disasters, or political disruptions, can act as external factors that disrupt implementation processes [17, 39, 40]. This reaffirms the experiences shared by the municipal employees.

### The inner setting

Municipal employees reported that all but two municipalities assigned reform implementation solely to the health and care sector, which they deemed vulnerable. A recent study showed that central decision-making and bureaucracy slowed down implementation [41]. Contrarily, two larger municipalities demonstrated broader collaboration. This aligns with research indicating that decision implementation efficacy improves with increased departmental and sectoral involvement in the decision-making process [36, 42].

Aside from one financially stable municipality, a shortage of personnel significantly hindered reform implementation. As illustrated in CFIR, chronic understaffing and high turnover hinder implementation, while sufficient resources enhance readiness for innovations [17]. Municipalities also faced coordination gaps between sectors, with poor inter-organizational communication posing a barrier. In contrast, strong intra-organizational communication supported front-line decision-making and facilitated implementation [36].

### Individual characteristics

A key challenge was the lack of municipal leadership, noted in CFIR as an important factor for successful implementation through networking, resource allocation and prioritization [17]. Leadership commitment and active participation strengthen the implementation climate and enhance effectiveness [43]. However, municipal employees attributed progress in the reform’s five key areas to dedicated champions or opinion leaders rather than strategic leadership. Opinion leaders influence attitudes and behaviors during implementation [36] and play a key role in providing knowledge, problem-solving, skill-building, and ensuring program continuity [20, 44]. Participants reported constraints in fulfilling reform roles due to limited personnel, time, and knowledge. Implementation success depends on individual competence, availability, time, and autonomy, as emphasized in CFIR-informed studies [45].

While this study focuses on municipal employees, a related study highlights the important role of older adults’ individual characteristics in implementing aging-in-place policies [46]. Traits like 'independence literacy’, shared responsibility with municipalities, and varying individual needs influence governance and decision-making. Older adults engaging in shared responsibility can foster collaboration, while unrealistic expectations of autonomy or insufficient municipal support may create challenges. A holistic approach, integrating perspectives from both employees and older adults, is essential for inclusive, locally tailored policies that address inequality and improve outcomes [46].

### The implementation process

Despite efforts from the national and regional support system, municipal employees, who engage with older individuals daily in their clinical practice, felt overlooked in terms of information sharing, and a clear sense of responsibility for the reform was lacking. Considering the significance of the reform's cross-sectoral foundation, Edmondson (2012) suggests that fostering collaborative teamwork, instead of solely relying on a ‘hero model’ where motivated individuals carry out most of the work, is crucial for enduring change [47]. As noted in CFIR-informed studies, building coalitions and promoting teamwork are commonly recommended strategies to tackle implementation barriers [44]. Yet, no municipalities reported conducting assessments or planning for reform focus areas, making the work seem arbitrary. Assessing the needs of both the recipients and those providing the service [45], as well as evaluating the context [25], are key factors in achieving successful implementation and ensuring equity in the implementation process.

Lastly, participants noted a decrease in enthusiasm for the reform during its implementation, possibly due to a change in government and the anticipation of a forthcoming reform. Engaging deliverers and recipients of an innovation is an often-overlooked part of implementation [48], but doing so helps ensure sustained change [49, 50]. Thus, it is vital to pinpoint and frequently engage with both deliverers and recipients from the early stages [51]. Successful implementation is more likely when advocates outnumber opponents and are strategically positioned, as noted in CFIR-informed studies [36].

### Interaction between CFIR domains and implications for implementation

The findings demonstrate how CFIR domains interact to shape reform implementation. Gaps in leadership and cross-sector collaboration (inner setting) were exacerbated by insufficient guidelines to clarify the reform's key elements and roles (innovation characteristics). External pressures, such as funding constraints and the COVID-19 pandemic (outer setting), limited resources and shifted priorities, while individual-level factors, such as dedicated champions, partially mitigated these obstacles. Similar issues are reported internationally, where studies highlight barriers to aging-in-place policies, including decentralization, resource allocation, and governance differences in high-income countries [6, 7]. Norway’s decentralized governance and geographic diversity inherently add complexity, requiring tailored strategies. Top-down approaches should define program theory and roles (the what) while fostering cross-sector teamwork and leadership training (the how). Sustainable funding and regular context assessments are vital for adapting reforms to local needs and evolving conditions. These findings underscore the need to balance local autonomy with robust top-down support to address structural barriers, promote equity, and sustain long-term implementation.

### Strengths and limitations

In this study, qualitative data was collected from a variety of municipal employees with diverse professional backgrounds. These individuals, who represent frontline providers [52], come from six distinct municipalities in Norway, each with unique demographic, economic, and geographic challenges. However, potential selection bias may occur as those who agreed to participate may hold specific characteristics influencing their decision to participate.

While data from frontline leaders, such as team and department managers, were included, perspectives from higher-level municipal leaders responsible for governance are not covered in this publication, which may be seen as a limitation. Findings were not validated with participants through member checking, which might have strengthened the study's rigor.

The author, along with two colleagues (two physiotherapists and one nutritionist), conducted all interviews, bringing expertise in geriatrics and implementation science. Using a responsive interviewing style [53], the researchers maintained an open stance, sustained eye contact, and mirrored interviewees' phrases [54]. The team engaged in ongoing dialogue, challenging 'assumed' truths in the data. A limitation may be that the study provides a single-time-point evaluation, limiting insights into the evolution of barriers, facilitators, and strategies during scaling up [55]. However, the detailed methodology and context descriptions enable readers to draw parallels to similar contexts, enhancing transferability [56].

## Conclusions

This study highlights the complexities of implementing aging policy in municipal contexts, as highlighted by municipal employees involved in the implementation of an "aging in place" reform policy. It emphasizes the need for sustainable leadership, cross-sector collaboration, a well-structured preparation phase, and clearly defined roles and responsibilities. While bottom-up strategies enhance the reform’s relevance and credibility by incorporating local knowledge and engagement, the findings reveal that these must be complemented by tailored, top-down strategies. Such strategies should provide a clear program theory to highlight the intervention's innovative aspects, define roles, and establish practical frameworks that foster implementation, build capacity, and ensure long-term sustainability. By addressing key barriers such as insufficient leadership, fragmented teamwork, and inadequate adaptation to local contexts, this study provides actionable insights for policymakers and practitioners to design reforms that are both context-sensitive and resilient to external disruptions.

## Data Availability

Due to restrictions in the main study's data collection approval, the data and materials for this manuscript are not available for sharing.

## Abbreviation

CFIR: The Consolidated Framework for Implementation Research
